# Using household survey data to explore the effects of the domiciliary environment on weight at birth: a multilevel mixed-effects analysis of the 2016 Ethiopian Demographic Health Survey

**DOI:** 10.1186/s12884-023-05521-9

**Published:** 2023-03-20

**Authors:** Aiggan Tamene, Aklilu Habte, Mihretu Tagesse, Zablon Wale Sewalem, Abel Afework

**Affiliations:** 1 School of Public Health, College of Medicine and Health Sciences, Wachemo University, Hosaena, Ethiopia; 2 Department of Clinical and Psychosocial Epidemiology, University of Groningen, Groningen, Netherlands; 3 Department of Environmental Health, College of Medicine and Health Sciences, Dilla University, Addis Ababa, Ethiopia

**Keywords:** Low birth weight, Demographic health survey, Domiciliary environment, Ethiopia

## Abstract

**Background:**

Low birth weight (LBW) is associated with infant mortality and postpartum health complications. In previous studies, overall LBW has been found to be significantly associated with several sociodemographic factors, including ethnicity, maternal age, and family income. Few studies have evaluated the association between environmental risk factors and LBW rates. This study investigated the effect of pre-birth water, sanitation, and hygiene (WASH) and housing conditions on self-reported low birth weight.

**Methods:**

The Ethiopian Demographic and Health Survey, which covered all administrative regions of Ethiopia from January to June 2016, provided data for this study. STATA version 16 was used to analyze 12,125 participants across weighted samples. Multivariable multilevel mixed-effect logistic regression analysis was conducted to determine the effects of each factor on the outcome while accounting for data clustering. The adjusted odds ratios and corresponding 95% confidence intervals were used to determine the statistical significance of the independent variables.

**Results:**

One thousand five hundred and seventeen newborns, or 12.59% [95% CI (10.2- 15.3)], had low birth weights. When other factors were taken into account, the following factors were significantly associated with low birth weight: not using small-scale water treatment technology before using water [AOR (95% CI) 1.36 (1.08–2.23)], burning solid fuels for energy [AOR (95% CI) 1.99 (1.60–2.21)], living in homes with natural wall coverings [AOR (95% CI) 1.81 (1.47–2.21)], using a shared latrine within a woman's housing complex or compound [AOR (95% CI) 1.63(1.06–2.25)], and living in peripheral, isolated regions [AOR (95% CI) 1.38 (1.06–2.21)].

**Conclusion:**

A little more than one out of every ten deliveries in Ethiopia was under normal (recommended) weight. This study shows that poor housing conditions and lack of household WASH infrastructure are independent predictors of poor birth outcomes among Ethiopian women, adding to the limited evidence that environmental factors within the domicile contribute to low birth weight. Interventions to address these issues may help lower the prevalence of LBW.

## Introduction

According to the World Health Organization (WHO), low birth weight (LBW) is defined as a weight of < 2500 g at birth. LBW is also a major cause of death among children under the age of five [1]. The long-term effects of LBW on postnatal development may include an increased risk of respiratory distress, infection, hypoglycemia, polycythemia, intellectual difficulties, cerebellar palsy, vision and hearing loss, and feeding and digestion issues [2–4].

It is well established that socioeconomic, demographic, and genetic factors affect LBW. In previous studies, race, maternal age and health, and family socioeconomic status were individual-level variables associated with LBW incidence [5–7]. It has also been recognized that several environmental factors increase the incidence of LBW. These elements include exposure to toxins in the air, water, and pesticides, as well as the proximity of the parent’s home to other environmental dangers including motorways and gas drilling sites [8, 9].

Nevertheless, the majority of prior studies on the environmental influences of LBW rates mostly concentrate on commonly observed air pollutants, such as gases (NOx, SO_2_, CO, and O_3_) and particulate matter (PM_10_ and PM_2.5_) [2, 10, 11]. Goal three of the Sustainable Development Goals (SDGs) states that "preventable deaths of infants and children under the age of five must end and maternal mortality must be reduced by 75 percent by 2030 [12]. Frameworks that have gained traction for reducing feto-maternal mortality, such as the "Three Delays" and "Continuum of Care" models, almost entirely concentrate on increasing access to and the quality of maternal health services, with little attention paid to broader environmental influences [13, 14].

Between sixty and eighty percent of communicable infections in Ethiopia are caused by poor hygiene, a lack of access to clean water, and other problems. Additionally, it's estimated that environmental conditions including overcrowding, a lack of access to water sources, and unsanitary facilities are responsible for 50% of the effects of undernutrition [15]. Only 17% of people in a given community practice improved hygiene, 65% of families had access to improved water sources, and 6.3% had improved sanitation [16]. The above-mentioned direct and indirect effects of WASH on health can be used to infer the plausibility of this effect on child health, but there is a lack of methodical mapping and evaluation of these numerous, complicated, and frequently overlapping pathways. Anecdotal evidence and observational studies make up the majority of the evidence for these pathways, which is generally weak.

According to previous studies, a person's health cannot be entirely explained by factors at the individual level; therefore, a novel methodology is needed to understand the community-level causal pathways of public health outcomes [17]. In the current study, we propose that examinations of these multi-level domiciliary conditions, which can have a significant impact on embryo-fetal development, should be conducted to better understand how the environment affects human reproduction.

Thus, the goal of this study was to present epidemiologic data on the potential effects of the various environmental risk factors in the home on the weight of newborns in Ethiopia. Determining whether household environment-related stress—both physical and psychosocial—affects birth outcomes for women in low-income countries is critical for understanding whether the global prevalence of LBW could be reduced by improving the environmental conditions in which pregnant women seek clean water and improved sanitation. Moreover, estimating the contribution of these risk factors to poor health could serve as the foundation for identifying and utilizing policy, advocacy, and programming synergies that will result in more effective, efficient, and equitable investments in both reproductive and environmental health sectors. Our objective was to address these research gaps, which would increase policy coherence and result in more effective interventions.

## Methods

### Study design and area

The 2016 Ethiopian Demography and Health Survey (EDHS) provided secondary data that were analyzed. Amhara, Benishangul-Gumuz, Gambella, Harari, Oromia, Somali, and the Southern Nations, Nationalities, and People's Region (SNNPR), together with two administrative cities (Addis Ababa and Dire-Dawa), participated in the Demographic and Health Survey. There are currently 115,114,480 people living in Ethiopia, according to the latest available United Nations statistics [18].

### Data source and study period

The data for this investigation were obtained from the official database of the Demography Health Survey (DHS) program; www.measuredhs.com after permission was requested. The Demographic and Health Survey in Ethiopia was conducted between 18 January and 27 June 2016.

### Sampling procedure, study population, and sample size

The 2007 Population and Housing Census, which used a stratified two-stage cluster sampling technique, served as the sampling frame for EDHS 2016. In the first stage, 645 enumeration areas (EAs) were carefully selected using a probability proportional to the size of the EA and an independent selection in each sampling stratum (202 in urban areas and 443 in rural areas). The second step involved the systematic selection of 28 households. The EDHS 2016 report includes a publication on the sampling methodology [19].

In this survey, mothers were asked to provide information on the birth weight of any child delivered within the previous five years. In addition, data on housing characteristics were gathered from the de jure population. A weighted sample of 19,318 women revealed that at least one birth had occurred in the five years before the study. The analysis was subsequently discontinued for 4828 mothers whose infants had not been weighed. A total of 699 mothers were excluded because they did not know the birth weights of their children. The final sample was a weighted sample of 12,125 participants.

### Study variables

#### Outcome variables

Low birth weight, defined as the weight of a newborn less than 2.5 kg at birth regardless of gestational age, was the study's outcome variable. All mothers who were interviewed between the ages of 15 and 49 who had given birth and whose children had been weighed and recalled were included. Newborns were classified into non-LBW birth weight ≥ 2,500 g = 0 and birth weight < 2,500 g = 1.

#### Independent variables

Potential risk factors of LBW were extracted from the data set after examining recent literature. The extracted variables were categorized as individual, household, and community-level variables because the 2016 EDHS data are hierarchical. Maternal factors that were unique to each woman were classified as individual-level variables (Table 1).

**Table 1:** Individual level variables extracted from EDHS 2016 data set for studying factors associated with LBW

Variable	Description	Category
Age	The age of the woman in years at the time of the survey	0. 15–24 1. 25–34 2. 35–49
Women education	The highest educational level attained at the time of the survey	0. No formal education 1. Primary education 2. Secondary education 3. higher education

On the other hand, household-level variables included characteristics that apply to all mothers residing in the same household (Table 2). The variables at the community level were those that applied to all women living in the same community (cluster), such as place of residence, region, community (cluster) poverty, and community women's education (Table 3). Individual factors within the cluster were aggregated to produce variables, such as community women's education and community poverty. Based on the national median values of the created variables, the generated variables were further divided into low and high categories.

**Table 2:** Household- level variables extracted from EDHS 2016 data set for studying factors associated with LBW

Variable	Description	Category
**Household wealth**	Scores were given to households based on the number and kinds of consumer goods they owned	0. Poorest 1. Poor 2. Middle 3. Rich 4. Richest
**Family Size**	The number of people who lived in the mother’s house	0. < 3 1. 4–7 2. > 7
**Type toilet** [18]	It is divided into two categories: improved (any non-shared toilet of the following types: flush/pour flush toilets to piped sewer systems, septic tanks, and pit latrines, ventilated improved pit [VIP] latrines, pit latrines with slabs, and composting toilets); and unimproved (shared toilet, flush/pour flush not to sewer/septic tank/pit latrine, pit latrine without slab/open pit, hanging latrine and others)	0. Improved 1. Unimproved
**Drinking water source** [19]	Listed as either unimproved sources (such as an unprotected dug well and spring, a tanker truck or cart with a small tank, and surface water) or improved sources (such as piped water, public taps, standpipes, tube wells, boreholes, protected dug wells and springs, and bottled water)	0. Improved 1. Unimproved
**Location of water source**	The location of the drinking water source	0. In dwelling/yard 1. Elsewhere
**Share toilet with other home/s**	Whether toilets are shared between a group of households in a single building or plot	0.No 1. Yes
**Water collection time** [19]	How long it takes to get water	0. < 30 min 1. > 30 min
**Person fetching water**	Water fetcher, conducted for off-premises sources	0. Adult female 1. Adult male 2. Child < 15 years 3. Other
**Place where family members wash hands**	Location of a hand washing facility	0. Fixed place 1. Mobile place 2. Not indwelling
**Essential agents**	Presence of water and/or detergents near handwashing stations	0. Yes 1.No
**Water treatment**	Filtering, boiling, adding bleach, and solar disinfection (SODIS) were considered to be effective water treatment techniques	0.No 1. Yes
**Service continuity**	Water service interruption for a full day or more over the last 14 days	0.No 1. Yes
**Disposal of child faeces**	Ways of *disposing faeces*	0. Left open 1. Rinse in the ditch 2. Garbage 3. Burry 4. Rinse in the toilet
**Cooking fuel** [20]	A new category called "clean fuel" was created by combining the categories of electricity, liquefied petroleum gas (LPG), natural gas, and biogas. As "solid fuel," items such as coal/lignite, charcoal, wood, straw/shrub/grass, crops, and animal manure were merged	0. Solid fuel 1. Clean fuel
**Flooring type** [21]	**Natural** [Earth, sand, clay, mud, dung] **Rudimentary** [Tablets/wood planks, Palm, bamboo, Mat, Adobe] **Finished** [Parquet, polished wood, Vinyl, asphalt strips, floor mat, Linoleum, Ceramic tiles, mosaic, Cement, Carpet, Stone, Bricks]	0. Natural 1. Rudimentary 2. Finished
**Wall type** [21]	**Natural** [No wall, Cane/palm/trunks Grass/thatch/palm leaf, Dirt, Mud and sticks, Tin/cardboard/paper/ bags, Thatched/straw] **Rudimentary [**Bamboo with mud, Stone with mud, uncovered Adobe, Plywood, Cardboard, Reused wood, Trunks with mud, Un-burnt bricks, Un-burnt bricks with plaster, Un-burnt bricks with mud] **Finished** [Cement, Stone with lime/cement, Bricks, Cement blocks, Covered adobe, Wood planks/shingles, Burnt bricks with cement]	0. Natural 1. Rudimentary 2. Finished
**Roof type** [21]	**Natural** [No roof, sod, straw, Grass/thatch/palm leaf] **Rudimentary** [Rustic mat, palm/bamboo, wood planks, cardboard, tarpaulin, plastic] **Finished** [Metal, wood, cement, ceramic tiles, roofing shingles, calamine/cement fibber, slate roofing sheet]	0. Natural 1. Rudimentary 2. Finished

**Table 3:** Community- level variables extracted from EDHS 2016 data set for studying factors associated with LBW

Variable	Description	Category
**Place of residence**	The geographic area of residence at the time of the survey	0. Urban 1. Rural
**Region**	Three categories were created: a small peripheral, a larger center, and a metropolitan area. Small periphery regions were characterized as Afar, Somalia, Benishangul, and Gambella The SNNPRs, Tigray, Amhara, and Oromia were categorized as major central areas. The administrative cities of Addis Ababa, Dire Dawa, and the Harari region make up the metro areas	0. Metro 1. Large central 2. Small peripheral
**Community Poverty** [22]	Described as the percentage of women who lived in the cluster's poorest homes. The cluster's overall poverty can be determined by adding up the individual households with the lowest wealth indices If clusters contained more poor households than the national median proportion (30%), they were classified as high; if not, they were classified as low	0. Low 1. High
**Community Education **[23]	Described as the percentage of women in the cluster who attended primary, secondary, or higher education. The sum of each woman's primary, secondary, and higher education levels might reveal the cluster of women's overall educational achievement It was labelled as high if a cluster had a primary/secondary/higher education proportion greater than or equal to the national median proportion (7.7%), or low if not	0. Low 1. High

### Data management and statistical analysis

Before the analysis, the variables were sorted, cleaned, and recorded using STATA version 14. Households whose outcome variables were missing or were marked as unavailable because their values could not be used were excluded. These data were recorded into the database under a special code designated "na" that either indicated responses that were judged to be inconsistent with other responses on the questionnaire and thought to be likely errors or answers with the value "Don't know."

### Multilevel analysis

Analytical and descriptive statistics including frequencies and proportions were computed. A weighted analysis was carried out to account for the uneven likelihood of selection between the strata caused by the non-proportional distribution of samples to different regions, residences, and study participants' non-response rates. Multilevel (three-level) regression was utilized because of the hierarchical structure of the 2016 EDHS data, where individuals nested within households and households nested within clusters. Biased parameter and standard error estimations could emerge from using single-level analysis while ignoring the hierarchical character of the data. Furthermore, hierarchical data do not support the normal logistic regression assumption of independent observations. By simultaneously analyzing the impacts of explanatory factors at various levels, multilevel analysis overcomes these constraints.

Variables with a *p*-value of less than 0.25 were included in the multivariable analysis following the bi-variable multilevel logistic regression analysis. The adjusted odds ratio was calculated with a matching 95% confidence level to show the strength of the association. The dependent variable was judged to be significantly associated with variables with a *p*-value ≤ 0.05. Using a Variance Inflation Factor (VIF) of < 10, multicollinearity between the individual- and community-level variables was examined.

### Random effects

Five random intercept models were fitted (Models 1, 2, 3, 4, and 5).

Model one (null model)—This model has only the intercept and no other independent variables. The intra-class correlation coefficient (ICC) and median odds ratio (MOR) statistics were calculated for the measures of variation (random effects). While MOR can measure unexplained cluster variability, ICC can explain cluster variability (heterogeneity). The ICC was 17%, indicating that variations in the prevalence of LBW were caused by variations at the cluster level. Significant group-level variance is shown by an ICC of at least 2%, which is a requirement for a multilevel study design. Additionally, intra-household clustering was suggestive of employing a three-level model as opposed to a two-level model that overlooks heterogeneity at the household level (controlling for intra-household and intra-cluster variability). Individual-, household-, and community-level variables were taken into account in Models 2, 3, and 4, respectively.

Model five was adjusted for the individual-, household-, and community-level factors. This model demonstrated that unobserved community- and household-level factors might account for 17.3% of the unexplained variation. The fifth model with the lowest deviance was selected as the best-fit model after comparison. The goodness of fit was also tested using Schwarz's Bayesian Information Criterion (BIC) and Akaike's information criterion (AIC). The lowest AIC or BIC values were deemed a better explanatory model.

## Result

### Socio-demographic characteristics

Mothers between the ages of 25 and 34 made up 77.1% of the study subjects. Similarly, more than half (63.7%) of the households had four to seven members. One-third (29%) of the mothers in the study had no formal schooling. Concerning household wealth, 17.89% of households were in the two lower quintiles. In terms of the participants' community-level characteristics, half (50.4%) of the participants lived in rural areas. Moreover, three fourth (78%) of the homes were located in large central areas. More impoverished households were observed in the majority (84.51%) of clusters compared to the national median proportion (Table 4).

**Table 4:** Distribution of individual-level and community-level socio-demographic factors, analysis from the 2016 EDHS, (weighted *n* = 12,125)

Variable	Frequency	Percentage
**Maternal age**		
15–24	3088	25.47
25–34	6,923	57.10
34–49	2,114	17.43
**Maternal education**		
No formal education	3,526	29.08
Primary education	4,562	37.62
Secondary education	2,188	18.04
Higher education	1,850	15.26
**Household wealth**		
Poorest	788	6.50
Poorer	1,256	10.36
Middle	1,697	13.99
Richer	1,901	15.68
Richest	6,484	53.48
**Family Size**		
< 3	2,734	22.55
4–7	7,735	63.79
> 7	1,656	13.66
**Place of residence**		
Urban	5,972	49.26
Rural	6,152	50.74
**Region**		
Metro^a^	1,944	16.04
Small Peripheral^b^	624	5.15
Large Central^c^	9,556	78.81
**Community Poverty**		
Low	10,247	84.51
High	1,878	15.49
**Community Education**		
Low	5,998	49.47
High	6,127	50.53

^a^Harari region, Dire Dawa, and Addis Ababa administrative cites

^b^Afar, Somalia, Benishangul, and Gambella

^c^Tigray, Amhara, Oromia, and the SNNPR

### Water, sanitation, and hygiene related characteristics of households

The majority of the households (71.85%) used improved water sources, whereas 66.9% had improved sanitation facilities. A large majority (97.36%) of households used water sources that were not in their own homes. Toilets were shared with another home in 44.09% of the surveyed households. Adult females collected water in three-quarters of the homes. Eighteen percent of the households had to travel for more than 30 min to collect water. A mobile handwashing station was present in 62% of the households. Soap or detergents were only present in places where individuals cleansed their hands in 33% of households.

Similarly, only 14.13% of homes treated their water at the point of use. A total of 61.96% of the households reported water service continuity lasting a full day or more in the 14 days before the survey. Concerning child feces, only 1.08% of the homes in the survey flushed child feces into the toilet (Table 5).

**Table 5:** Distribution of household-level water, sanitation, and hygiene-related factors, analysis from the 2016 EDHS, (weighted *n* = 12,125)

Variable	Frequency	Percentage
**Type of toilet facility**		
Unimproved	8,113	66.91
Improved	4,012	33.09
**Water source**		
Unimproved	3,412	28.15
Improved	8,712	71.85
**Location of water source**		
In own dwelling/yard	110	1.64
Elsewhere	6,610	98.36
**Share toilet with other home/s**		
Yes	4,635	44.09
No	5,978	55.91
**Water collection time**		
< 30 min	9,852	81.25
> 30 min	2,273	18.75
**Person fetching water**		
Adult female	5,219	78.33
Adult male	490	7.36
Child < 15 years	852	12.78
Other	102	1.53
**Place where members wash hands**		
Fixed place	979	8.07
Mobile place	7,566	62.40
Not observed/not indwelling	3,580	29.53
**Essential agents near the handwashing station**^a^		
Yes	2,827	33.16
No	5,697	66.84
**Effective water treatment**		
Yes	1,714	14.13
No	10,411	85.87
**Water service continuity (last 14 days)**		
Yes, Interrupted for a full day or more	5,352	61.96
No, not interrupted for a full day	3,285	38.04
**Disposal of child faeces**		
Left open	942	13.46
Put/rinsed in the ditch	3,086	54.37
Throw it into the garbage	1,305	19.64
Burry	801	11.44
Put/rinsed in the toilet	76	1.08

^a^soap/detergents observed near the handwashing station

### Household fuel and housing material-related characteristics of households

In the current study, 82% of the households used solid fuels to meet their energy needs, and slightly more than half (56.5%) of the households cooked meals in a different building from where they slept and lived. Regarding the construction materials used in homes, the survey found that natural flooring materials were used in 55.7% of the homes and that rudimentary finishing was used on the majority of the walls (81.94%). For roofs, 78.63% of the homes used improved materials (Table 6).

**Table 6:** Distribution of household characteristics and housing material-related factors, analysis from the 2016 EDHS, (weighted *n* = 12,050)

Variable	Frequency	Percentage
**Type of cooking fuel**		
Solid fuel	9,883	82.01
Clean fuel	2,168	17.99
**Place of cooking**		
In the house	3,792	31.47
In a separate building	6,813	56.54
Outdoors	1,445	11.99
**Household floor type**		
Natural	6,720	55.76
Rudimentary	125	1.04
Finished	5,206	43.20
**Household wall type**		
Natural	318	2.64
Rudimentary	9.874	81.94
Finished	1,858	15.42
**Household roof type**		
Natural	2,211	18.35
Rudimentary	365	3.03
Finished	9,475	78.63

### Prevalence of low birth weight

A total of 1517 infants, [(12.59%, 95% CI (10.2- 15.3)], were born underweight in the five years before the survey.

### Factors associated with LBW

#### The measure of association (fixed effects)

Utilizing solid fuels, having unimproved wall covering, and not using point-of-use water treatment were found to be strongly associated with LBW when considering household-level factors. As per community-level characteristics, LBW was found to be significantly associated with living in small peripheral states in the multilevel multivariable mixed effects logistic regression analysis. When other factors were taken into account, households that did not employ small-scale water treatment technology before using water had a 1.3 times higher likelihood of having babies who were underweight at birth [AOR (95% CI) 1.36 (1.08–2.23)].

The likelihood of neonates having low birth weight was also significantly associated with families using solid fuels for energy [AOR (95% CI) 1.99 (1.60–2.21)]. Infants with low birth weight were 1.8 times more likely to be delivered in dwellings where the walls were coated with natural materials [AOR (95% CI) 1.81 (1.47–2.21)]. Using a shared sanitary facility within a woman's domicile or compound was also associated with a greater risk of low birth weight [AOR (95% CI) 1.63(1.06–2.25)]. Compared to metro inhabitants, those who lived in small peripheral regions were 1.3 times more likely to give birth to neonates who were below normal weight [AOR (95% CI) 1.38 (1.06–2.21)] (Table 7).

**Table 7:** Multilevel multivariable logistic regression analysis of the prevalence of low birth weight and associated factors, analysis from the 2016 EDHS, (weighted *n* = *12,125*)

Variable categories	Model-II (AOR) (95% CI)	Model-III (AOR) (95% CI)	Model-IV (AOR) (95% CI)	Model-V (AOR) (95% CI)
**Maternal education**				
No formal education	1.89 (1.06–3.05)*			1.51 (0.811–2.81)
Primary education	1.22 (0.72–2.05)			1.10 (0.64–1.90)
Secondary education	0.94 (0.52–1.70)			0.90(0.49–1.64)
Higher education	1			1
**Water source**				
Unimproved		1.59 (1.11–1.99)*		0.87(0.57–1.36)
Improved		1		1
**Water treatment**				
Yes		1		1
No		1.55 (1.05–2.52)*		1.36 (1.08–2.23)*
**Share toilet with other homes**				
No		1		1
Yes		1.84 (1.16–2.04)		1.63(1.06–2.25)*
**Type of cooking fuel**				
Unclean fuel		1.44 (1.04–2.19)*		1.99 (1.60–2.21)*
Clean fuel		1		1
**Household wall type**				
Natural		2.50 (1.12–5.55)*		1.81 (1.47–2.21)*
Rudimentary		1.36 (0.92–2.03)		1.13 (0.73–1.750
Finished		1		1
**Region**				
Small Peripheral			1.41 (0.95–2.08)	1.38 (1.06–2.21)*
Large Central			1.60 (1.05–2.44)*	1.25 (0.81–1.93)
Metro			1	1
**Community Education**				
High			1.44 (1.03–2.01)*	0.94 (0.62–1.43)
Low			1	1

^*^statistically significant at *p*-value <0.05

#### Measures of variations (random effects)

The ICC in the empty model demonstrated that the variation in the prevalence of LBW across clusters accounted for 17% of the overall variation. Furthermore, when choosing two places at random, the odds ratio between the highest risk area and the lowest risk area was 1.60 (95% CI: 1.26, 1.93). Nine percent of the national variation observed in the empty model was explained by individual, household, and community-level variables (PCV = 9%). When compared to the empty model, individual-, household-, and community-only models, and then again to the entire model, the values of AIC, BIC, and Deviance all indicated a continuous decrease. This demonstrates that the final model developed during the analysis had acceptable goodness of fit (Table 8).

**Table 8:** Random intercept variances and model fit statistics comparison of three-level mixed effect logistic regression model, analysis from the 2016 EDHS, (weighted *n* = 12,125)

Measures	Null Model (Model 1)	Model-II	Model-III	Model-IV	Model V
**Random effects**					
ICC	0.17	0.25	0.162	0.165	0.173
PCV	Ref	0.80	0.89	0.84	0.90
MOR	1.62	1.64	1.60	1.58	1.60
AIC	1149.63	1148.311	1150.552	1147.164	1125.108
BIC	1160.489	1186.204	1188.446	1174.231	1155.482
**Model fitness**					
Deviance	1144.26	1134.3	1136.55	1137.1641	1129.10

## Discussion

The WHO estimates that poor WASH and housing conditions account for over ten percent of the world's illness burden when multiple health consequences are taken into account. However, the impact of inadequate housing and WASH on prenatal birth weight has not yet been quantified [24]. According to the latest available WHO statistics, Ethiopia experienced 28,020 LBW fatalities in 2020, accounting for 4.97% of all deaths. The country is ranked 37^th^ in the world for LBW mortality, with an age-adjusted death rate of 9.52 per 100,000 people [25]. As a result, many argue that frameworks that have been popular for lowering feto-maternal mortality have missed opportunities for potential synergies between newborn and environmental health.

The major objective of this study was to identify multilevel characteristics associated with LBW in Ethiopia. Accordingly, the analysis found that factors evolving from individual, household, and community (cluster) levels had an impact on weight at birth. The likelihood of LBW varied significantly between households and clusters. Because of this heterogeneity, it was inferred that individual-level factors might not account for the entire variation. The results revealed that in the five years before the survey, 12.59% [95%CI (10.2- 15.3)] of newborns were delivered with low birth weight. This implies that Ethiopia still struggles with preventable events that might be averted by integrating prenatal health with housing and WASH interventions.

There is a growing body of evidence, supported by biological plausibility, which indicates that poor water quality affects fetal and neonatal outcomes in a variety of ways [26, 27]. Water contamination by microbiological pathogens, chemicals, and radiation is a problem that affects the health of a significant segment of the population [20, 28]. This study has provided quantifiable measures of association by identifying that households that did not employ point-of-use water treatment before using water had a 36% higher likelihood of having babies who were underweight at birth.

Household water treatment (HWT), also known as point-of-use water treatment, offers a technique to reduce risk levels by treating water that has been contaminated at the source and by domestic handling in communities without consistent access to safe drinking water [29]. Although it has been demonstrated that the opportunity and financial costs of acquiring and treating water consume a sizable portion of the resources of poor families [20], little is known about how and why some people modify their behavior concerning water treatment while others do not. Future studies must gain a deeper understanding of the factors driving water treatment decisions.

Ninety-four percent of Ethiopian families use solid fuels like wood, kerosene, and charcoal for cooking or heating [21]. Traditional domestic energy practices have a significant impact on socioeconomic development, the environment, and human health [30]. The total burden of disease is five times greater than that caused by outdoor air pollution. Women are disproportionately exposed to pollution from cooking fuels because they are typically in charge of childcare and cooking [30].

According to earlier studies, exposure to home air pollution during pregnancy can endanger both the mother and the unborn child. Carbon monoxide, the primary component of incomplete combustion, reacts with hemoglobin to generate carboxyhemoglobin, resulting in a diminished ability to carry oxygen to tissues and the growing fetus [31]. As a result, increased prenatal fatalities occur and newborns have low birth weights. The rates of electrification in urban and rural areas in Ethiopia are drastically different. Clean cooking fuel is primarily used by urbanized households with highly educated household heads [32]. In order to strengthen their capacity to motivate community members across a wide variety of user types or levels of readiness to use clean fuels and to provide social support for such behaviors, future interventions should consider these factors.

Historical data from the United States and Europe support the theory that housing modifications have an impact on feto-maternal outcomes [33, 34]. Regarding long-term health metrics, those who live in completed homes, with brick or plaster walls, and a tiled roof perform much better than people who live in the least desirable type of housing [35]. Poor housing quality is linked to a higher risk of developing many illnesses, most notably respiratory and infectious diseases, but also blood pressure and cardiovascular disease [36].

In the present study, infants with low birth weights were more likely to be born in homes covered in natural cover than in those with finished walls. Given the rapid development currently occurring in sub-Saharan Africa, a full understanding of the protective efficacy of housing modifications is required to calculate the potential reductions in fetal morbidity and death. As a result, the Ethiopian government will need to work with the private sector to encourage housing development by increasing access to and upgrading the quality of the available stock, while also making it simpler for people to access land and housing finance.

Research has shown that public toilets are a vital component of building sustainable, open, and inclusive communities. However, human feces is a major source of transmission for many common diseases, as well as many vaginal and urinary infections, which makes it crucial to offer hygienic public toilets [37]. Today’s modern public health discourse, however, questions whether public toilets act as a conduit for pathogen transmission and physical disability or as a facilitator of health [38]. In many parts of the developing world, women are forced to navigate difficult—and perhaps personally dangerous—social and environmental public conditions to locate a safe, private area to defecate and bathe, which creates psychosocial stress [39]. Additionally, the absence of household WASH infrastructure exposes women to criminality and harassment, which have previously been identified as important predictors of women's vulnerability to LBW [28].

Few studies have specifically examined the influence of shared toilets on adverse pregnancy outcomes. Those that did, report that using a shared toilet was more likely to result in low birth weight than using a private family restroom [37, 40]. This was further illustrated in the current study, which found that mothers who reported sharing toilets with other households had a 63% higher likelihood of giving birth to underweight newborns. Interventions that introduce gender-sensitive sanitation — including clean, safe, and separate toilets, and access to water—may thus serve to ensure gender-equitable living conditions that address the needs of all people, including pregnant women in Ethiopia.

People who lived in small peripheral regions were more likely to give birth to neonates who were below normal weight than metro residents. The Ethiopian government has recognized the small peripheral regions Afar, Somalia, Benishangul, and Gambella as developing regional states because of the high prevalence of poverty and social metrics that trail far below national averages [41]. While variations in socio-demographic variables may account for regional variations in prevalence, it is known that deficiencies, inconsistent service delivery, inequity, and inefficiency are some of the key fetal health challenges. Thus, for such regions to deliver the goods and services that the native population desires with a comparative or absolute advantage, more work must be done to ensure the development of the human and social capital of the regions.

This study reveals that a lack of household WASH infrastructure is a risk factor for poor birth outcomes in women in low-income countries, adding to the scant evidence about environmental causes of LBW. To the best of our knowledge, this study is the first to quantify the proportional contributions of the individual-, family-, and community-level environmental factors that impact birth weight in Ethiopia. Despite the aforementioned benefits, this study has some limitations. Because the data were cross-sectional, they were inadequate for determining causal correlations. The lack of temporal data is unlikely to introduce any bias; however, major changes in housing standards and WASH services are unlikely to occur over such short periods. Another potential limitation of this study is the lack of information on housing features other than the building materials used to construct floors, walls, and roofs in the survey data. There is no information available about a home's interiors, such as whether the eaves are open or whether any windows or doors have screens. Finally, a recall bias may be introduced when measuring historical events.

## Conclusion

The intricate interactions between access to water, sanitation, and hygiene as well as housing conditions and birth outcomes among Ethiopian women were examined in this study. The study also provides opportunities for further research and insights that may have policy- and program-related implications for environmental and child-health programs. Important risk factors for LBW include households without a private source of drinking water, living in small peripheral regions, using solid fuels as the main source of energy, and households in which women handle their sanitary needs in shared or public spaces. Because of the multiple pathways through which poor access to water, sanitation, and hygiene may negatively impact newborn health outcomes, it is necessary to integrate WASH and housing considerations to support efforts within the newborn and child healthcare program in Ethiopia and vice versa to leverage greater progress in reducing neonatal mortality and morbidity.

## Data Availability

The datasets generated during and analyzed during the current study are not publicly available but are available from the corresponding author Aiggan Tamene via apublic22@gmail.com on reasonable request.
